# The Role of Natural Anthelmintics in Small Ruminant Health: A Global Narrative Review

**DOI:** 10.3390/ani16111653

**Published:** 2026-05-28

**Authors:** Tiziana Maria Mahayri, Alessia Serra, Filomena Dessì, Maria Piera Demontis, Maria Vittoria Varoni, Giuseppe Moniello, Giovanni Garippa

**Affiliations:** Department of Veterinary Medicine, University of Sassari, 07100 Sassari, Italya.serra29@phd.uniss.it (A.S.); f.dessi@studenti.uniss.it (F.D.); dpiera@uniss.it (M.P.D.); giovannigarippa@gmail.com (G.G.)

**Keywords:** small ruminants, gastrointestinal nematodes, productive efficiency, natural anthelmintics

## Abstract

Gastrointestinal nematodes (GINs) represent a major and persistent threat to the health and productivity of small ruminants. Increasing anthelmintic resistance has made traditional drugs less effective, requiring sustainable alternatives. Among the most promising solutions are natural anthelmintics: bioactive compounds and organisms derived from plants, essential oils, condensed tannins, saponins, probiotics, and nematophagous fungi. These natural agents are not just supplementary but vital for integrated parasite control. This review examines the host–parasite relationship, focusing on resistance, tolerance, and resilience, and discusses the impact of GINs on productivity. It evaluates natural anthelmintics, including their mechanisms, efficacy, benefits, and safety. To ensure the successful integration of natural anthelmintics into mainstream parasite control programs, future research must prioritize real-world validation, longitudinal safety assessments, and comprehensive cost–benefit analyses.

## Simple Summary

Parasitic worms in the digestive system are a major problem for sheep and goats because they harm animal health and reduce farm productivity. Farmers have traditionally used chemical drugs to control these parasites, but many worms are becoming resistant to these treatments. This review explores natural alternatives that may help control parasites, including substances from plants, plant oils, beneficial microbes, and fungi that attack parasite larvae. It also explains how animals respond to infections and how these natural methods may reduce parasite levels and support animal health.

## 1. Introduction

Small ruminants, particularly sheep and goats, play an important role in the farming sector [1]. Their ability to convert low-quality forage into valuable products, such as milk, meat, fiber, and skins, makes them essential contributors to food security [2]. The global demand for small ruminant products has increased recently, due to changes in dietary preferences and the increasing recognition of goat milk as an alternative to cow milk [3].

However, parasitic infections remain among the most prevalent health challenges in grazing animals, significantly impairing their productivity [4]. Gastrointestinal nematode (GIN) infection constitutes one of the major limitations in goat and sheep production [5]. While conventional anthelmintic drugs have constituted the primary therapeutic strategy, their frequent administration and misuse have led to the emergence of anthelmintic resistance (AR) in nearly all major classes of drugs [6]. Consequently, there is an urgent need to explore alternative, sustainable, and eco-friendly methods of parasite control [7]. Among these alternatives, plant- and fungi-derived bioactive compounds have emerged as potential natural anthelmintic agents [8]. Their appeal lies in their potential to act through multiple mechanisms, reducing the risk of resistance development while supporting animal health in a more holistic manner [9]. Many of these compounds may offer additional benefits such as immune modulation, antioxidant effects, and improved ruminal function [10]. Despite growing interest, however, the integration of natural anthelmintics into gastrointestinal nematode control programs remains limited, largely due to variability in efficacy and a lack of standardization in application protocols [11].

What is notably lacking is not the identification of candidate compounds, but a rigorous, comparative, and translationally oriented evaluation of these agents under biologically relevant conditions. Existing reviews tend to focus on single classes of natural products in isolation, thereby overlooking critical differences in mechanisms of action and consistency of efficacy.

To address these challenges, this review focuses on natural anthelmintics that have demonstrated in vivo efficacy against major gastrointestinal nematodes in sheep and goats. Unlike previous reviews that focused on single classes of natural anthelmintics, it provides a comparative evaluation across multiple classes, including plant metabolites, essential oils, nematophagous fungi, and microbial metabolites, emphasizing translational relevance and practical applicability.

## 2. Methodology

This narrative review was conducted between October 2025 and February 2026, following structured principles for transparent and reproducible literature synthesis in accordance with the SANRA recommendations [12]. Before evaluating natural anthelmintic agents, the host–parasite relationship and the effects of gastrointestinal nematodes (GINs) on productivity are discussed, providing essential context for the study.

### 2.1. Literature Search Strategy

A literature search was carried out using electronic databases and search engines. The papers were found in PubMed, Scopus, Google Scholar and Web of Science. The search covered studies published between 2000 and 2026. To identify relevant articles, search terms were used including combinations of keywords related to gastrointestinal nematodes and natural control strategies in small ruminants, such as “gastrointestinal nematodes”, “GIN”, “sheep”, “goats”, “small ruminants”, “plant extracts”, “phytochemicals”, “bioactive compounds”, “secondary metabolites”, “botanical compounds”, “phytogenic compounds”, “essential oils”, “biological control”, “nematophagous fungi”, and “natural anthelmintics”. Boolean operators (AND, OR) were used to find the aforementioned terms.

### 2.2. Eligibility Criteria

The inclusion criteria for the studies were developed based on the study’s objectives. As a result, the studies were included if they (1) investigated natural compounds or biological agents targeting gastrointestinal nematodes in sheep or goats, (2) reported in vivo, in vitro, or field-based efficacy, and (3) were original research articles or relevant review papers. Exclusion criteria were: (1) studies not related to helminth control, (2) studies focusing on non-ruminants, and (3) articles lacking primary data.

When multiple studies addressed similar compounds, priority was given to those providing in vivo or field-based evidence.

### 2.3. Study Selection, Quality Assessment and Data Extraction

**Figure 1:**
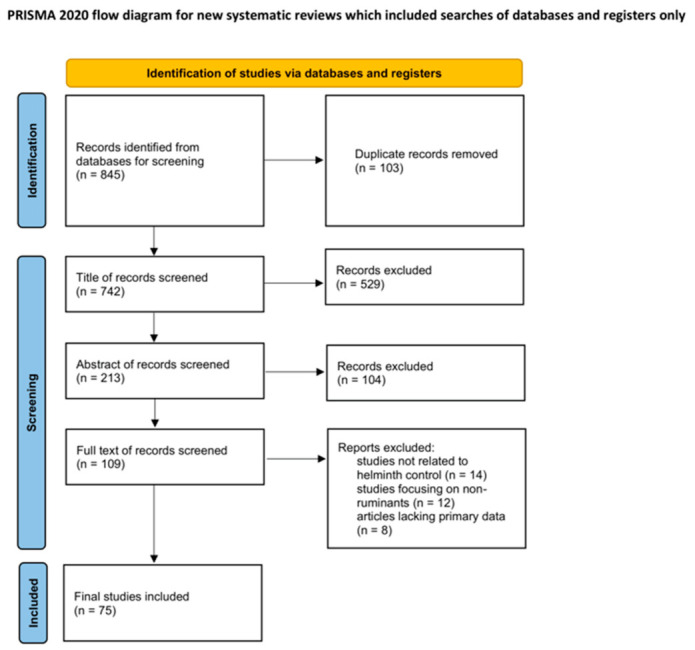
PRISMA flow diagram of the study selection and screening process.

Study selection was conducted by two independent reviewers, and discrepancies were resolved through discussion and consensus. Relevant studies were screened based on titles and abstracts, followed by full-text evaluation where appropriate. A total of 75 studies met the inclusion criteria and were included in the review, as indicated by the PRISMA flowchart [13] (Figure 1). Although no formal risk-of-bias scoring system was applied, this is consistent with the narrative nature of this review. Each study underwent a structured qualitative appraisal. Selection was predicated on methodological rigor, including the robustness of the experimental design, adequacy of sample sizes, the clinical relevance of the infection models, and the internal consistency of the reported outcomes. The following information was collected from each selected study: type of intervention, parasite species, study design (in vitro, in vivo, field trial), and the reported efficacy outcomes.

### 2.4. Approach to Evidence Synthesis

Given the heterogeneity of study designs, interventions, and outcome measures, a quantitative meta-analysis was not feasible. Therefore, a qualitative synthesis approach was adopted. Evidence was critically appraised and organized according to the level of biological validation (in vitro vs. in vivo vs. field conditions), the reproducibility and consistency of findings, and the translational relevance under practical farming conditions. Greater interpretative weight was assigned to in vivo and field-based studies compared to in vitro findings, which were considered exploratory.

## 3. Relation Between Host and Parasite

The relationship between hosts and parasites must be taken into consideration since gastrointestinal parasitic infections in small ruminants continue to rise [14].

While parasites adapt to survive within their hosts, hosts employ protective strategies such as resistance, tolerance, and resilience to defend themselves. Resistance is described as the ability to limit the number of parasites hosted or their rate of reproduction [15]. The interaction between host and parasite depends on the nutritional state, immune system and genetic variants [16]. Tolerance is explained as the capability to maintain performance while being infected [17]. In this case, the immune responses tend to repair the damaged tissues and involve immunological mechanisms against the toxins produced by the parasites rather than the parasite itself [18]. Resilience is defined as the capacity to tolerate parasites without developing clinical symptoms [19] and is related to tolerance, since it is also defined as the ability to preserve undiminished performance levels during infection [20].

Concerning parasites, the two basic notions are infectivity and pathogenicity. Different studies have shown that infectivity, which is the ability to infect a host, varies according to the strain of parasite [21]; it is also expressed differently depending on the breed of the host [22]. Pathogenicity, which is the ability to cause disease, may also vary depending on the parasite species and on the strain variability within each species [21].

## 4. Impact of Gastrointestinal Nematodes on Small Ruminant Performance and Economics

Small ruminant production is negatively affected by parasitic infection [14], which leads to important economic losses and the emergence of Anthelmintic resistance (AR).

### 4.1. Influence of Parasitism on Animal Performance

Gastrointestinal nematodes (GINs) are a major factor that negatively impacts the performance of small ruminants, leading to reduced productivity across various sectors, including weight gain, milk production, wool production, and reproductive efficiency [23]. One of the most direct and noticeable effects of GIN infection in small ruminants is a reduction in weight gain, particularly in young animals [24]. This leads to slower growth rates, delayed time to market, and higher feed costs as animals take longer to reach the optimal weight for sale [25]. Milk yield in dairy sheep and goats could also be affected, reducing both the quantity and quality of the milk produced [26]. In dairy production systems, the effects are often most apparent during the lactation period, as the infection diverts energy from milk production toward combating the parasite burden [27]. For wool-producing sheep, GINs can severely impact wool quality and quantity. The parasitic burden disrupts the animal’s overall health, leading to weaker, poorer-quality wool fibers and reduced fleece yield [28]. GINs also affect reproductive efficiency in small ruminants, particularly in ewes and does [29]. Altogether, the cumulative effects of GIN infections significantly compromise the overall performance of small ruminants, undermining productivity across growth, lactation, wool yield, and reproduction.

### 4.2. Overall Economic Losses Due to Parasitic Infection

Gastrointestinal nematodes are one of the parasitic infections that have the greatest economic impact on small ruminant production worldwide, particularly in Europe [30]. The majority of studies have focused on evaluating the reduction in weight, milk, wool, and reproductive efficiency to quantify the losses in productivity. However, research concerning the overall economic losses due to GIN infection on a global scale remains relatively scarce [31]. In Europe, the annual estimated costs of helminth infections are substantial and considerable. For example, in dairy sheep, the economic losses due to parasitism are estimated to be around €151 million annually. For the ovine meat sector, losses are estimated at €206 million, and for dairy goats, the annual cost is around €86 million. However, these estimates are often imprecise due to data gaps and limitations in the current models, as well as regional variations in parasite prevalence and control practices. It is important to note that these costs only account for direct losses and do not fully reflect the broader economic consequences of GIN infections, such as the impact on the agricultural economy, food security, and livelihoods of small-scale farmers [31].

## 5. Natural Anthelmintics

The concern of anthelmintic resistance to conventional drugs such as benzimidazoles and macrocyclic lactones [32] and their high cost have stimulated the search for alternative strategies for controlling gastrointestinal parasites, including the use of plant extracts [33,34,35,36,37,38], essential oil extracts [32,39,40,41], nematophagous fungi [42,43], and microbial metabolites [44,45,46]. Given the heterogeneity of these approaches and the wide range of experimental models used to evaluate their efficacy, a structured overview is important to integrate the available evidence.

To ensure consistency across heterogeneous interventions, each category is discussed by distinguishing the in vitro evidence, in vivo efficacy, and translational constraints relevant to practical implementation.

### 5.1. Plants

Plants and their bioactive compounds have long been investigated as alternatives or complements to conventional anthelmintics. Secondary metabolites may act against gastrointestinal nematodes (GINs) through multiple mechanisms, including the disruption of larval development, inhibition of egg hatching, or direct toxicity to adult worms. Research includes in vitro and in vivo studies, as well as field trials, though the translation of laboratory findings into practical farm applications remains a key challenge. Despite the range of published data, overall efficacy remains highly variable. Differences are observed not only between plant species but also among extracts derived from the same species, underscoring the importance of methodological aspects. Factors influencing efficacy include plant part used [47], extraction method [47], compound concentration [48], and nematode species targeted [49].

Numerous studies were based on in vitro assays assessing ovicidal, larvicidal, or motility-inhibiting effects [50]. Plant extracts including *Artemisia herbaalba*, *Anacardium occidentale* shell, *Artocarpus heterophyllus*, and *Artocarpus camansi* have shown important in vitro antiparasitic activity, sometimes achieving near-complete inhibition of egg hatching or larval migration [48,51,52,53]. However, the interpretation of these results requires caution because in vitro assays do not account for key physiological constraints including rumen fermentation and animal metabolism. This may lead to an overestimation of the anthelmintic potential, especially when high concentrations are used in the studies.

On the other hand, in vivo evidence remains limited, with more moderate effects. While high in vitro inhibition (84–100%) was shown for some plant extracts, such as *Artemisia campestris* and grape pomace, lower efficacy was demonstrated when they were introduced in vivo. For instance, 50 mL of macerated *Artemisia campestris*, orally administered to sheep as a single dose, showed only a 20% reduction in FEC [54]. Grape pomace, rich in saponins, flavonoids, and tannins, demonstrated a 61% FEC reduction in *Haemonchus contortus* larvae when included as a 20% partial replacement of the concentrate feed [55]. Dose, duration, and type of preparation and administration induce variations; for example, 50 mL of *P. granatum* macerate administered as a single dose to sheep led to a 50% reduction in FEC [54], while the administration of *P. granatum* peel extract (PPE) at a dose of 200 mg/kg for 7 days led to a 97% reduction in FEC in ruminants, including sheep and goats [33]. Also, tannins appear to depend strongly on dose and administration strategy. Dietary supplementation with quebracho tannins in sheep experimentally infected with gastrointestinal nematodes produced only moderate reductions in fecal egg counts and worm burdens, with efficacy remaining limited under low-concentration feed exposure [56,57]. In addition, quebracho tannins worked more quickly and more effectively against *H. contortus* when administered as drench; *T. colubriformis* may require a higher dose or a prolonged exposure to the drench [56]. These differences likely reflect the substantially higher concentration of hydrolyzable tannins, gallic acid, and ellagitannins in concentrated peel extracts compared with crude aqueous macerates. Repeated dosing may also prolong nematode exposure to active metabolites and improve efficacy against surviving larval stages and reinfections. Additionally, extraction solvent and concentration strongly influence phytochemical recovery and bioavailability.

Azadirachta indica administered as a crude powder and crude methanolic extracts at the doses of 1 and 3 g/kg of body weight to sheep has shown a maximum reduction in FEC by 29.3% and 40.2%, respectively, against *Haemonchus contortus* and *Trichostrongylus* species [58]. On the other hand, papaya latex has shown strong efficacy, with a reduction of 98% in FEC in sheep infected with *Haemonchus contortus* and *Trichostrongylus colubriformis* [59]. Similarly, *T. portulacastrum* and *M. paradisiaca* revealed an in vivo reduction of 85.6% and 80.7% in FEC against *Haemonchus contortus* when administered to sheep at 8.0 g/kg [60].

Some other plant extracts did not show significant effects in vivo. Aqueous extracts of *Salix caprea*, despite their salicylate content, showed negligible in vivo efficacy when given at 50 mL to sheep as a single dose [54]. In addition, the mixture of *Arbutus unedo* L., *Pistacia lentiscus* L., and *Quercus ilex* L. showed no significant reduction in FEC in naturally infected adult sheep [61].

Indirect host-supportive effects have also been noted, for example, pomegranate supplementation has been associated with measurable productivity benefits, including increased milk yield, indicating potential dual antiparasitic and nutritional functionality [62].

Plant anthelmintic activity is mainly related to the composition of secondary metabolites present in its extracts. Several classes of phytochemicals including tannins, flavonoids, phenolic acids, alkaloids, triterpenoids, limonoids, acetogenins, and proteolytic enzymes have shown anthelmintic activity against gastrointestinal nematodes [48]. Through multiple mechanisms, these compounds can interfere with parasite physiology and lead to an inhibition of egg hatching, impairment of larval motility, oxidative stress, and direct toxicity against adult worms [63].

Tannins are the most investigated phytochemical class in ruminant helminth control [64]. Both condensed tannins and hydrolyzable tannins have demonstrated inhibitory activity against several developmental stages of nematodes including eggs, infective larvae, and adults [65,66]. Mechanistically, tannins may bind to glycoproteins present on the nematode cuticle and digestive tract, altering membrane permeability and nutrient uptake. In addition, tannins reduce parasite development within the host gastrointestinal tract [66]. Antiparasitic efficacy seems to depend not only on total tannin concentration but also on structural features including degree of polymerization and hydroxylation pattern. Larger tannin polymers and highly hydroxylated structures have been associated with stronger inhibition of larval motility [67,68].

*Punica granatum* is one of the most tannin-rich botanical anthelmintics [48]. Pomegranate peel extracts contain high concentrations of hydrolyzable tannins including punicalagin isomers and ellagic acid derivatives. These compounds contribute to ovicidal and larvicidal activity through protein precipitation and oxidative disruption of parasite tissues [33,69]. Hydroalcoholic extraction procedures generally recover higher concentrations of ellagitannins and phenolic compounds than crude aqueous preparations, potentially explaining the large variation in reported fecal egg count reduction among studies evaluating *P. granatum* formulations [70].

Similar observations have been reported for grape pomace extracts, where condensed tannins, flavonoids, and phenolic acids contribute to inhibit larval migration and reduce nematode viability [34]. Several other plant-derived compounds have demonstrated distinct mechanistic profiles. In *Anacardium occidentale* shell extracts, anacardic acids, cardanol, and cardol have been associated with larval paralysis and the inhibition of egg hatching [51]. Similarly, *Annona muricata* contains annonaceous acetogenins, alkaloids, and phenolic constituents capable of inhibiting egg hatching and impairing parasite survival [71]. Azadirachtin from *Azadirachta indica* has shown an effect on growth and reproductive processes in helminths, although in vivo efficacy was moderate and mainly dependent on dosage and extract preparation [58].

The anthelmintic activity of Papaya latex has been primarily attributed to cysteine proteases including papain, chymopapain, caricain, and glycyl endopeptidases [72]. These proteolytic enzymes hydrolyze structural proteins of the nematode cuticle, resulting in loss of cuticular integrity and parasite death. Papaya latex demonstrates a relatively well-defined biochemical mechanism involving the enzymatic degradation of parasite tissues. This mechanistic specificity may partly explain its comparatively high in vivo efficacy against *Haemonchus contortus* and *Trichostrongylus colubriformis* [73,74].

**Figure 2:**
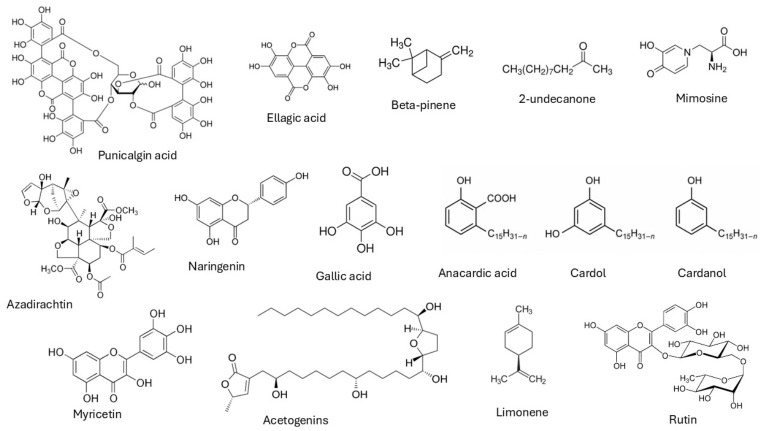
Chemical structures of principal secondary metabolites with anthelmintic activity against gastrointestinal nematodes.

Considerable phytochemical variability also exists within the same botanical species. Environmental stress, soil composition, seasonal variation, geographical origin, genotype, harvesting period, and extraction methodology may substantially alter the concentration and relative abundance of active metabolites [75]. Such variability is particularly important when comparing studies using crude extracts, powdered plant materials, aqueous macerates, hydroalcoholic extracts, or enriched fractions. A major limitation across the available literature is the insufficient chemical characterization and standardization of tested botanical preparations. Some studies report antiparasitic efficacy without quantitative phytochemical and metabolomic profiling, preventing an accurate comparison between studies and limiting reproducibility [33,54]. Figure 2 illustrates the chemical structures of the main bioactive constituents involved in plant anthelmintic activity.

Most botanical extracts are intrinsically multi-component systems containing numerous bioactive metabolites. Therefore, enhanced efficacy observed in polyherbal formulations may result from additive or synergistic interactions among phytochemicals derived from different plant species. For example, compounds containing both condensed tannins and polyphenols may affect multiple stages of the nematode life cycle, reducing the risk of resistance development [76]. The selection of plant-based interventions should therefore consider both the spectrum of activity and potential additive or synergistic effects, particularly in integrated parasite management programs.

The main limitation of plant-based anthelmintics lies in the gap between experimental efficacy and practical applicability. Most studies have been conducted under controlled experimental conditions, limiting extrapolation to commercial production systems [76]. Standardization of active compounds is also an unresolved issue. In addition, dose reproducibility and timing of administration should be systematically evaluated. Differences between single-dose and repeated treatments as well as variations in preparation methods influence outcomes [33,54]. Long-term safety data are scarce, and issues related to palatability, voluntary intake, and seasonal variation in phytochemical composition remain insufficiently addressed [77]. Limited safety and toxicity data, particularly for long-term use, further constrain their application. These limitations help to explain the slow progression of plant-derived anthelmintics toward routine on-farm implementation, despite promising biological activity. Practical feasibility and consistency remain key barriers to wider implementation. From a translational perspective, a subset of plant-derived interventions, such as Punica granatum and grape pomace, shows potential for integration into parasite control programs, particularly when incorporated into the diet of small ruminants. However, their efficacy remains moderate and variable, as several factors still need to be standardized and controlled. Consequently, their role is currently best considered complementary rather than substitutive within integrated parasite management strategies.

Table 1 summarizes the anthelmintic activity of the plants reported across previous studies.

### 5.2. Essential Oils

Essential oils (EOs) have also been investigated for their anthelmintic activity against different development stages of gastrointestinal parasites [32,39,41,82]. Similar to plant extracts, EO activity has been evaluated across in vitro assays [83], in vivo studies, and, to a lesser extent, under field conditions.

Most of the available data have been obtained from in vitro experiments. Essential oils from *Origanum vulgare*, *Foeniculum vulgare*, *Tagetes patula* and *Satureja* spp. have shown nearly complete egg hatch inhibition of the usual gastrointestinal nematodes [32,84]. Oils from *Rosmarinus officinalis* have demonstrated activity against gastrointestinal nematodes, while those from *Lavandula angustifolia* and *Quercus infectoria* have shown similar effects at concentrations of 1–50 mg/mL against *Marshallagia marshalli* including reduced egg hatching, inhibition of larval motility, induction of DNA damage, and decreased larval viability [85,86]. However, as with other plant-derived compounds, these results must be interpreted cautiously, as in vitro systems do not replicate the complexity of the gastrointestinal environment including rumen metabolism, host absorption, and compound biotransformation. High concentrations commonly used in vitro may further inflate apparent efficacy.

In vivo evidence remains limited and generally indicates more moderate effects. In addition, several essential oils with strong in vitro effects lack in vivo validation, limiting their translational relevance. *Thymus vulgaris* have shown modest reductions in fecal egg counts (approximately 25%) when administered orally to sheep at a dose of 100 mg/kg as a single dose, despite strong in vitro activity [32]. Similarly, *Cymbopogon citratus* essential oil, which demonstrated high in vitro efficacy, achieved only limited reductions in fecal egg counts (23–47%) when administered orally for three days at doses of 450–500 mg/kg in sheep [39]. Essential oils from *Lippia sidoides*, administered to sheep at 230 and 283 mg/kg as a single dose, showed variable effects, with fecal egg count reductions ranging from 30% to 56% [40]. Finally, *Mentha* × *piperita* has shown moderate in vivo efficacy, with fecal egg count reductions ranging from approximately 26% to 46% when administered at 150 mg/kg as a single dose, suggesting some biological activity but insufficient consistency for standalone application [87].

Variation exists in the concentrations used across studies, as well as in treatment regimens. Some studies used single-dose administration, while others applied repeated treatments over several days, making direct comparisons difficult [32,39]. Higher doses or prolonged administration may enhance efficacy, but also raise concerns regarding toxicity, cost, and practical feasibility under field conditions. In addition, differences in formulation further complicate the interpretation of dose–response relationships. This lack of standardization in dosing strategies remains a major limitation for reproducibility and practical application. The volatility and rapid degradation of essential oil components may further reduce their bioavailability in vivo, particularly in ruminants where rumen fermentation can alter or inactivate active compounds. Formulation strategies such as encapsulation or nanoemulsification have been proposed to improve stability and delivery, but current data remain insufficient to demonstrate consistent benefits under practical conditions [39].

The main bioactive constituents responsible for the anthelmintic activity of essential oils are volatile secondary metabolites, especially carvacrol, thymol, anethole, p-cymene, and γ-terpinene [32]. These compounds act by reducing parasite motility, decreasing egg hatching [32,39,40,41,84,86], disrupting parasite cell membranes, and inducing oxidative stress [85].

**Figure 3:**
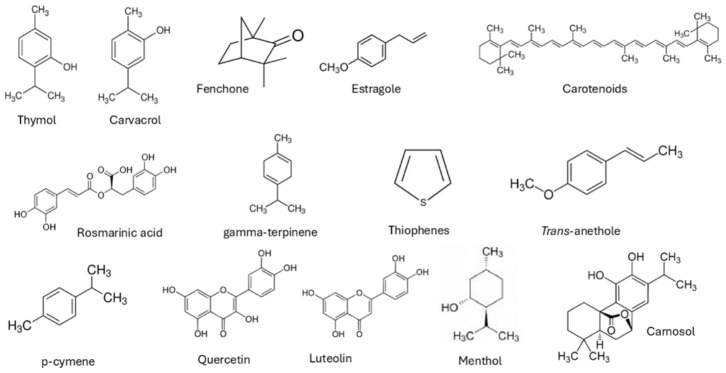
Chemical structures of major bioactive constituents identified in anthelmintic essential oils.

Phenolic monoterpenes such as carvacrol and thymol, abundant in *Thymus vulgaris*, *Origanum vulgare*, *Satureja hortensis*, *Satureja montana*, and *Lippia sidoides* are among the most extensively studied constituents [88]. These compounds impair cell membrane, increase ion permeability, and interfere with neuromuscular signaling, leading to paralysis and nematode mortality [89,90]. Their strong lipophilicity facilitates rapid penetration of the nematode cuticle, explaining the high in vitro ovicidal and larvicidal activity frequently reported (>90–100% egg-hatch inhibition). However, their volatility and rapid metabolism in the rumen may limit in vivo persistence and reduce efficacy under field conditions [40,91]. Other monoterpenes such as linalool, linalyl acetate, terpinen-4-ol, and 1,8-cineole, present in *Lavandula angustifolia*, exhibit additional mechanisms including oxidative stress induction, DNA damage, and inhibition of larval motility. These compounds may act synergistically within the EO matrix, enhancing antiparasitic potency [85]. Aromatic compounds such as trans-anethole, estragole, and fenchone from *Foeniculum vulgare* demonstrate strong ovicidal activity, likely through enzyme inhibition and interference with embryonic development [32]. Similarly, thiophenes and carotenoids from *Tagetes patula* have been associated with larval paralysis and cuticular disruption [84]. Also, the essential oil of *Mentha* × *piperita*, rich in menthol, menthone, menthyl acetate, and 1,8-cineole, exerts its anthelmintic activity through membrane destabilization, neuromuscular interference, and oxidative stress, contributing to the moderate in vivo reductions in egg hatching and fecal egg counts reported in peppermint-based studies [87]. Figure 3 illustrates the chemical structures of the main bioactive constituents involved in the anthelmintic activity of essential oils.

Similar to plant extracts, multi-component EOs often outperform single-compound formulations, likely due to synergistic effects on different stages of the parasite life cycle, reducing the risk of resistance development. Selection of EOs for parasite management should therefore consider both the spectrum of activity and potential additive or synergistic interactions, particularly within integrated parasite management programs [92].

Despite promising activity, most studies have been conducted under controlled experimental conditions, limiting direct translation to farm-scale application. Key barriers include variability in EO composition due to plant origin, extraction method, and seasonal factors as well as palatability, dosage consistency, and long-term safety data, which remain insufficiently addressed [93].

Table 2 summarizes the anthelmintic activity of the essential oils reported across previous studies.

### 5.3. Nematophagous Fungi

Nematophagous fungi (NF) can also be used to control gastrointestinal nematode infections in small ruminants by targeting the free-living stages of parasites in feces and pasture rather than the parasitic stages within the host [98]. Nematophagous fungi are soil microorganisms that live as sporophytes and can transform their mycelia into capture organs, specifically designed to capture and destroy nematodes. Their predatory activity is supported by the ability to produce various metabolites with nematicidal activity [99]. Their activity is mediated through mechanical capture, secretion of proteolytic enzymes, and in some species, the production of lectins that interfere with nematode development [100,101]. Through these mechanisms, NF can immobilize larvae, degrade the nematode cuticle and eggshell, inhibit larval development, and ultimately reduce pasture contamination [102]. Several classes, including serine proteases, chitinases, lipases and lectins, have demonstrated strong antagonistic activity against nematodes. Mechanistically, these compounds interfere with parasite physiology by digesting structural proteins of the cuticle, degrading egg layers, disrupting membrane integrity, and inducing oxidative stress, leading to larval mortality [103,104].

Most available data come from in vitro studies, in which several fungal species have demonstrated strong antagonistic activity against nematodes. For example, *Arthrobotrys musiformis*, *Aleuria aurantia*, *Marasmius oreades*, *Coprinopsis cinerea*, *Pochonia chlamydosporia*, and *Monacrosporium thaumasium* have shown predatory activity, nematicidal effects, developmental inhibition (approximately 80–95%), and the ability to attach to and destroy eggs in vitro [100,101]. Despite these promising results, the lack of in vivo validation for most of these species substantially limits their practical relevance. As with other biological control agents, in vitro efficacy does not necessarily predict field performance due to environmental variability and ecological interactions.

In contrast, *Duddingtonia flagrans* is the most extensively studied and the only nematophagous fungus with substantial in vivo evidence supporting its efficacy. This species produces chlamydospores capable of surviving passage through the gastrointestinal tract of ruminants [105]. These chlamydospores are highly resistant structures, protected by multilayered, melanized walls that prevent degradation by digestive enzymes, pH fluctuations, and mechanical abrasion during transit through the gut [106]. Once excreted in feces, these spores germinate and form trapping structures that capture and destroy L3 larvae, thereby reducing pasture infectivity. Both in vitro and in vivo studies have demonstrated significant reductions in larval populations and fecal egg counts with predatory activity [107]. In one study, lyophilized *D. flagrans* preparations were given to sheep at different concentrations (1 × 10^6^, 5 × 10^6^, and 1 × 10^7^ chlamydospores/kg body weight) as a single dose. No negative effects on key clinical parameters were observed, and the treatment significantly reduced the number of developing larvae in feces, with an efficacy of up to 92.99% [108]. In another study, goat kids and lambs were administered 5 × 10^5^ chlamydospores/kg body weight daily for three months during pasture season. Goat kids showed a 58% reduction in fecal egg counts; however lambs showed no significant reduction [109]. In a further study, periparturient ewes treated orally with *D. flagrans* at a dose of 1 × 10^6^ chlamydospores/kg body weight every three days, from 15 days before lambing until 42 days postpartum, exhibited a 97.4% reduction in fecal egg counts [110].

Considerable variability exists across studies in terms of dosage, frequency of administration, and duration of treatment. Protocols range from single-dose applications to repeated or continuous administration over several weeks or months, making direct comparisons difficult [108,109,110]. Sustained delivery, such as daily or periodic dosing throughout the grazing period, appears to be more effective than single administrations, as it ensures continuous fungal presence in feces and prolonged larval suppression. However, higher doses and long-term administration raise practical considerations, including cost, labor, and compliance under farm conditions.

Overall, nematophagous fungi offer a biologically rational and environmentally compatible approach to nematode control in small ruminants. However, most species remain constrained by laboratory-only evidence. Advancing NF toward routine use will require robust in vivo validation, standardized formulations, and field-scale assessments to ensure consistent efficacy under grazing conditions and to support their integration into sustainable parasite management programs.

Table 3 summarizes the anthelmintic activity of the nematophagous fungi reported across previous studies.

### 5.4. Microbial Metabolites

Microbial metabolites represent a heterogeneous class of alternative anthelmintic strategies including both antiparasitic metabolites and host-mediated resilience enhancers. Bacterial culture filtrates derived from abomasal isolates have shown remarkable in vitro activity against *H. contortus*. The study relied on the serial dilution of abomasal contents, isolation of bacterial strains via nutrient agar culture, and subsequent exposure of parasite eggs and larvae to cell-free supernatants. Under these conditions, 100% egg hatch inhibition and 6–100% larval or adult mortality have been reported [113]. These findings indicate potent direct antiparasitic effects. However, the absence of in vivo validation, lack of standardized dosing, and variability in bacterial isolates limit the translational relevance of these results.

Other microbial metabolites have demonstrated in vivo activity. *Bacillus thuringiensis* (Bt) crystal (Cry) proteins are the most mechanistically defined and experimentally advanced. A recent study using Cry5B formulations, administered to sheep as oral suspensions in water at three doses of 60 mg/kg, reported 90% reductions in fecal egg count and 75% reductions in adult worm burdens, supporting the translational potential of this approach [46]. However, despite strong efficacy, Bt-based interventions remain constrained by formulation stability, delivery efficiency, and regulatory uncertainty.

Probiotic organisms, including *Saccharomyces boulardii*, primarily exert direct and indirect effects. Supplementation at 40 mL/day containing ~1 × 10^8^ CFU/mL over a 26-day period in small ruminants has been associated with significant reductions in FEC and infective L3 larval counts. In addition, indirect effects have also been observed, including increased IgG levels and improved cellular immune responses [45]. These outcomes suggest immune modulation and improved gut barrier function as primary mechanisms rather than direct parasite toxicity. Similarly, multi-strain probiotic formulations, including bacterial species combined with *Saccharomyces* spp., administered at doses of approximately 5 g per lamb per day, have shown more nuanced effects. While consistent reductions in FEC were not observed, treated animals exhibited decreased female worm size and uterine egg content, improved fecal consistency, and markers of enhanced gut health [44]. Such findings support a role for probiotics in reducing parasite fecundity and improving animal performance, but they fall short of direct parasite control.

Despite promising biological activity, microbial metabolites face significant limitations. Many compounds remain preclinical or poorly standardized, and the direct antiparasitic activity of probiotics is inconsistent. Factors such as strain variability, formulation, dosage, duration, and administration route influence outcomes. Long-term safety data and practical feasibility under commercial farm conditions are limited, and regulatory approval processes further constrain use. These limitations explain the slow progression of microbial metabolites toward routine on-farm implementation.

From a translational perspective, Bt-derived crystal proteins currently stand out as the only microbial metabolites demonstrating reproducible direct in vivo efficacy, whereas probiotics primarily contribute host resilience and improved parasite fecundity management. Consequently, microbial metabolites are best considered complementary rather than substitutive tools within integrated parasite management programs.

Table 4 summarizes the anthelmintic activity of the microbial metabolites reported across previous studies.

## 6. Discussion

To better contextualize the relative performance of the different natural anthelmintic strategies, a comparative synthesis of their in vivo efficacy was conducted. Plant-derived compounds and essential oils represent the most extensively studied groups, with a wide range of efficacy outcomes; however, their performance is highly variable, reflecting differences in composition, dosing, and experimental design. Compared to essential oils, plant-derived extracts tend to show more stable but generally moderate in vivo efficacy, likely due to their integration into the diet and sustained exposure within the gastrointestinal tract [54]. Essential oils often demonstrate less consistent in vivo outcomes, which can be attributed to their volatility, rapid degradation, and limited bioavailability [115]. In contrast, nematophagous fungi, particularly *Duddingtonia flagrans*, demonstrate more consistent in vivo efficacy despite a smaller evidence base, likely due to their direct environmental mode of action, which bypasses host metabolism and avoids the bioavailability and degradation constraints that affect plant-derived compounds and essential oils [108,109,110]. Microbial metabolites occupy a distinct position, as their effects are often mediated through host modulation rather than direct parasite elimination, resulting in more moderate but biologically meaningful outcomes [44]. Overall, these differences emphasize that efficacy alone is not sufficient to determine practical value; rather, consistency, safety, and feasibility under field conditions are critical determinants of translational potential. This comparative perspective reinforces the need to prioritize interventions that combine reliable in vivo performance with realistic applicability in integrated parasite management systems.

## 7. Gap Analysis

Despite growing interest in natural anthelmintics as alternatives or complements to synthetic drugs in small ruminant systems, the field remains methodologically fragmented and underdeveloped [116]. Current research is characterized by experimental designs with variable extraction methods and changing dosing strategies that limit reproducibility and cross-study comparability [117]. As a result, despite a substantial volume of experimental work, the evidence base remains weak for translational applications.

A critical limitation lies in the poor translation of in vitro findings into in vivo efficacy. While numerous plant-derived compounds demonstrate activity against helminth eggs or larvae under controlled laboratory conditions, these effects are frequently attenuated or absent in live animals [54]. This discrepancy reflects the insufficient understanding of rumen metabolism, compound bioavailability, degradation kinetics, and host–parasite–microbiota interactions. Consequently, in vitro screening is often overinterpreted as evidence of practical anthelmintic potential.

Furthermore, the field lacks comprehensive dose–response, safety, and toxicity data. Most studies employ short-duration interventions, rarely assessing chronic exposure or potential negative effects on animal health and rumen function [87,116].

There are currently practical limitations in the availability and robustness of commercially applied natural anthelmintic products for small ruminants, as most alternatives remain at the level of experimental or niche field use rather than fully standardized veterinary interventions. The first is *Duddingtonia flagrans*, introduced in Brazil through the product Bioverm^®^ (GhenVet Saúde Animal, Paulínia, São Paulo, Brazil), representing a biological control strategy based on nematophagous fungi for gastrointestinal nematode management. Findings suggest that Bioverm^®^ can reduce dependence on chemical anthelmintics and contribute to managing resistance issues, supporting the practical relevance of *D. flagrans* as a biological control tool [118]. However, even in this case, its performance is strongly influenced by environmental conditions and management practices, and it is generally used as a part of integrated parasite control strategies rather than as a standalone solution.

Research efforts are also disproportionately concentrated on condensed tannins, particularly tannin-rich forages, while other natural anthelmintics such as microbial metabolites remain underexplored [119]. This narrow focus could lead to prioritizing some compounds without properly comparing their effectiveness, potentially overlooking more effective alternatives.

It should be noted that the efficacy of natural anthelmintics may differ between sheep and goats. Although some studies report comparable responses between the two hosts [33], biological differences can lead to a variability in outcomes. Goats generally exhibit a higher metabolic rate, which can affect the pharmacokinetics of administered compounds and may require higher effective doses [120]. In addition, goats tend to develop a weaker acquired immune response to gastrointestinal nematodes compared to sheep, potentially influencing treatment efficacy [121]. Differences in feeding behavior and parasite exposure patterns further contribute to variability in response. Consequently, results obtained in one species should not be directly extrapolated to the other without careful consideration, even when similar trends are observed within the same study.

In addition, the efficacy of natural anthelmintics was evaluated on its own rather than studied with targeted selective treatment, grazing management, or genetic resistance strategies. This limits their relevance to production systems, where multiple strategies are used together to reduce drug use and slow resistance development [122].

Finally, the absence of regulatory and translational pathways represents a structural challenge. Even when efficacy is demonstrated, few candidates progress beyond experimental trials, reflecting regulatory uncertainty, lack of standardized evaluation criteria, and limited engagement with farmers. This gap between academic research and practical use makes it hard for promising natural treatments to reach the farm or market. An overview of the major research gaps identified in the use of natural anthelmintics for gastrointestinal nematode control in small ruminants, together with their implications and future research needs, is summarized in Table 5.

## 8. Conclusions

This review provides a critical evaluation of natural anthelmintics in small ruminants by shifting the focus from experiments to biological and translational relevance. In contrast to the main literature, which is largely dominated by in vitro assays and analyses of individual compound classes, this work prioritizes in vivo evidence and integrates multiple categories of natural agents. A fundamental limitation is the persistent overestimation of efficacy based on laboratory data and the lack of standardization necessary for practical implementation. The analysis demonstrates that only a limited subset of natural anthelmintics currently meets the minimum criteria for reliability, consistency, and applicability under real farming conditions. By directly comparing diverse intervention strategies, including plant-derived compounds, essential oils, nematophagous fungi, and microbial metabolites, this review provides a realistic hierarchy of their effectiveness and readiness for integration into parasite management programs. Future research should prioritize standardized in vivo validation, long-term safety assessment, and the integration of natural anthelmintics within evidence-based parasite management frameworks.

## Figures and Tables

**Table 1:** Anthelmintic efficacy of plant extracts against gastrointestinal nematodes in ruminants.

Plant	Nematode Target	Bioactive Compounds	Evidence	Efficacy	Farm Applicability	References
*Punica granatum*	*Haemonchus* spp., *Ostertagia*, *Trichostrongylus*, *Cooperia*, *Nematodirus*, *Teladorsagia*, *Chabertia*	Punicalagin and ellagic acid	In vivo	50–97% FEC reduction; 15.5% increase in milk yield	Moderate—efficacy demonstrated but extract standardization and dose consistency remain limiting	[33,54,62]
*Artemisia campestris*	*Haemonchus*, *Trichostrongylus*, *Teladorsagia*, *Chabertia*	Beta-pinene, 2-undecanone, and limonene	In vitro and in vivo	20% FEC reduction; 100% egg hatch inhibition	Moderate—strong laboratory activity, modest field efficacy	[54,78]
*Artemisia herba-alba*	*Haemonchus contortus*	Phenolic compounds	In vitro	98.67% egg hatching inhibition	Low—no in vivo validation	[48]
*Salix caprea*	*Haemonchus*, *Trichostrongylus*, *Teladorsagia*, *Chabertia*	Phenolic compounds	In vivo	~0.1% FEC reduction	Low—biologically ineffective	[54]
*Annona muricata*	*Haemonchus contortus*	Annonaceous acetogenins, alkaloids and phenolic compounds	In vitro and in vivo	84–89% inhibition of eggs, L_3_, and adults; 42.5% FEC reduction	Moderate—strong laboratory activity, modest field efficacy	[71,79]
Grape pomace	*Haemonchus contortus*	Tannins, flavonoids and phenolic acids	In vitro and in vivo	100% larval migration inhibition; ~61% FEC reduction	Moderate—by-product availability and feed integration feasible but standardization and dose consistency remain limiting	[34,55]
*Anacardium occidentale shell*	*Haemonchus contortus*	Anacardic acid, cardol, and cardanol	In vitro	100% L_3_ paralysis; ~50% egg hatch inhibition	Low—no in vivo validation	[35,51]
*Leucaena leucocephala*	*Haemonchus contortus*, *Cooperia* spp., *Ostertagia* spp., *Chabertia* spp., *Moniezia* spp. *Strongyloides* spp.;	Mimosine and condensed tannins	In vitro and in vivo	~20% egg hatch inhibition; ~22% L_3_ inhibition; ~60% prevalence reduction	Moderate—nutritional trade-offs and toxicity require control	[80,81]
*Artocarpus heterophyllus*	*Trichostrongylus* spp., *Oesophagostomum* spp., *Haemonchus* spp. and *Bunostomum* spp. *Caenorhabditis elegans*	Condensed tannins	In vitro	90–100% L_4_ mortality	Low—no in vivo validation	[52,53]
*Artocarpus camansi*	*Caenorhabditis elegans*	Triterpenes, sterols and phenolics	In vitro	~90% L_4_ mortality	Very low—limited translational relevance	[53]
*Mentha pulegium*	*Haemonchus contortus*;	Flavonoids and tannins	In vitro and in vivo	60.4% FEC reduction; 71.6% worm burden reduction; 91.6% egg hatch inhibition	Moderate—extract standardization and dose consistency remain limiting	[37]
*Azadirachta indica*	*Haemonchus contortus*; *Trichostrongylus*	Azadirachtin	In vivo	29.3–40.2% FEC reduction	Moderate—extract standardization and dose consistency remain limiting	[58]
Papaya latex	*Haemonchus contortus*; *Trichostrongylus colubriformis*	Cysteine proteinases	In vivo	98% FEC reduction	High—strong efficacy demonstrated in vivo	[59]
*Trianthema portulacastrum*	*Haemonchus contortus*	Phenolic acids and flavonoids	In vivo	85.6% FEC reduction	High—strong efficacy demonstrated in vivo	[60]
*Musa paradisiaca*	*Haemonchus contortus*	Ellagic acid, gallic acid, rutin, myricetin and naringenin	In vivo		High—strong efficacy demonstrated in vivo	[60]
Quebracho tannin	*Trichostrongylus colubriformis*; *Haemonchus contortus*	Tannins	In vivo	30% FEC reduction	Moderate—moderate effect	[56,57]

**Table 2:** Anthelmintic efficacy of essential oils against gastrointestinal nematodes in ruminants.

Essential Oil	Nematode Target	Bioactive Compounds	Evidence	Efficacy	Farm Applicability	References
*Thymus vulgaris*	*Haemonchus contortus*, *Trichostrongylus*, *Teladorsagia*, *Chabertia*	Carvacrol and thymol	In vitro and in vivo	Egg hatching inhibition > 90%; larval development and motility ~90%; ~25% FEC reduction	Moderate—strong laboratory efficacy; limited field validation and standardization	[32,41,94]
*Origanum vulgare*	*Haemonchus*, *Trichostrongylus*, *Teladorsagia*, *Chabertia*	Carvacrol, thymol, rosmarinic acid and flavonoids	In vitro and in vivo	Egg hatching inhibition 100%, 43.2% and 60.1% FEC reduction	Moderate—demonstrated efficacy but dosing and palatability constraints	[32,95]
*Foeniculum vulgare*	*Haemonchus*, *Trichostrongylus*, *Teladorsagia*, *Chabertia*	Trans-anethole, fenchone, and estragole	In vitro	Egg hatching inhibition 100%	Low—no in vivo validation	[32]
*Satureja hortensis*	*Haemonchus*, *Trichostrongylus*, *Teladorsagia*, *Chabertia*	Phenolic monoterpenes (carvacrol, thymol, γ-terpinene, *p*-cymene), phenolic acids (rosmarinic acid) and flavonoids	In vitro	Egg hatching inhibition >99%	Low—no in vivo validation	[32]
*Satureja montana*	*Haemonchus*, *Trichostrongylus*, *Teladorsagia*, *Chabertia*	Carvacrol, thymol, p-cymene and γ-terpinene	In vitro	Egg hatching inhibition 100%	Low—no in vivo validation	[32,96]
*Cymbopogon citratus* (including nanoemulsion)	*Haemonchus contortus*	Terpenes, phenols, and flavonoids	In vivo and in vitro	~98% egg hatch inhibition; limited in vivo FEC reduction (23–47%)	Moderate—formulation improves activity, but field performance inconsistent	[39]
*Lippia sidoides*	*Haemonchus contortus*	Carvacrol, thymol and flavonoids (quercetin and luteolin)	In vivo	38–50% FEC reduction	Moderate—demonstrated efficacy but dosing and palatability constraints	[40]
*Tagetes patula*	*Haemonchus contortus*	Carotenoids (lutein, zeaxanthin), flavonoids (quercetin), thiophenes, and terpenoids	In vitro	100% egg hatch and larval inhibition	Low—no in vivo validation	[84]
*Rosmarinum officinalis*	Gastrointestinal nematodes	Phenolic acids, diterpenes, carnosic acid, carnosol, and rosmarinic acid	In vitro	97–100% egg hatch inhibition; 27–74% adult motility reduction	Low—no in vivo validation	[86]
*Lavandula angustifolia*	*Marshallagia marshalli*	Linalool, linalyl acetate, terpinen-4-ol 1,8-Cineole and (Eucalyptol)	In vitro	DNA damage, inhibition of motility (80.05%) and reduction in egg hatching	Low—no in vivo validation	[85]
*Quercus infectoria*	*Marshallagia marshalli*	Hydrolysable tannins, gallic acid, and ellagic acid	In vitro	DNA damage, inhibition of motility (80.05%) and reduction in egg hatching	Low—no in vivo validation	[85]
*Mentha* × *piperita* L.	*Trichostrongylus*, *Teladorsagia*, *Haemonchus*, *Chabertia*	Menthol, menthone, menthyl acetate and 1,8-Cineole (Eucalyptol)	In vivo and in vitro	Egg hatching reduction 20–90%, FEC reduction 26.86–46.04%	Moderate—in vivo efficacy confirmed, safe, standardization needed	[87]
*Coriandrum*	*Haemonchus contortus.*	Linalool, Geranyl acetate, Gamma-terpinene, Camphor	In vitro	86–100% egg hatching reduction	Low—no in vivo validation	[97]

**Table 3:** Anthelmintic efficacy of nematophagous fungi against gastrointestinal nematodes in ruminants.

Nematophagous Fungi	Nematode Target	Evidence	Efficacy	Farm Applicability	Reference
*Arthrobotrys musiformis*	*Haemonchus contortus*	In vitro	Predatory activity ~71%, nematicidal ~93%	Low—no in vivo validation	[43,111]
*Aleuria aurantia*	*Haemonchus contortus*	In vitro	Developmental inhibition (>95%)	Low—no in vivo validation	[101]
*Marasmius oreades*	*Haemonchus contortus*	In vitro	Developmental inhibition (>95%) and larval death	Low—no in vivo validation	[101]
*Coprinopsis cinerea*	*Haemonchus contortus*	In vitro	Developmental inhibition (>95%)	Low—no in vivo validation	[101]
*Duddingtonia flagrans*	*Trichuris* *trichiura*, *Haemonchus contortus*	In vivo and in vitro	L3 larvae destruction; predatory activity (~62%); significant EPG reduction in vivo	High—chlamydospores survive GI transit and reduce pasture contamination; practical on-farm application feasible but large-scale field validation needed	[100,112]
*Pochonia chlamydosporia*	*Trichuris* *trichiura Haemonchus contortus*	In vitro	Egg attachment and destruction; no L3 predation	Low—no in vivo validation	[100]
*Monacrosporium thaumasium*	*Trichuris* *trichiura Haemonchus contortus*	In vitro	L3 larval capture and destruction; egg attachment without destruction	Low—no in vivo validation	[100]

**Table 4:** Anthelmintic efficacy of microbial metabolites against gastrointestinal nematodes in ruminants.

Microbial Metabolites	Nematode Target	Evidence	Efficacy	Farm Applicability	Reference
*Bacillus thuringiensis* crystal protein	*Haemonchus contortus*	In vitro and in vivo	44–75% larval development reduction; 90% FEC reduction; 75% parasite burden reduction	Moderate—demonstrated in vivo efficacy but formulation and delivery remain limiting	[46,114]
Bacterial culture filtrates (abomasal isolates)	*Haemonchus contortus*	In vitro	100% egg hatch inhibition; 6–100% larval/adult mortality	Low—no in vivo validation	[113]
*Saccharomyces boulardii*	*Haemonchus contortus*	In vivo	Significant FEC reduction and L3 counts; boosted IgG, cellular immunity	Moderate—improves host resilience rather than direct anthelmintic activity	[45]
Multi-strain probiotic (bacterial + *Saccharomyces* spp.	*Haemonchus contortus*	In vivo	Reduced female worm uterine egg content and size; improved fecal consistency; no consistent FEC reduction	Low—effects mainly indirect and inconsistent	[44]

**Table 5:** Major gaps, consequences, and required research actions.

Identified Gap	Consequences	Research Direction
Various methodologies	Poor reproducibility; difficulty in comparing efficacy	Development of harmonized protocols (e.g., extraction methods, dose ranges, standardized bioassays)
Weak correlation between in vitro and in vivo outcomes	Overestimation of in vivo efficacy; limited field application	Studies that link rumen metabolism and host factors to bioactive effects; validated dose–response in live animals
Limited safety/toxicity assessment	Unknown risk to host health; barriers to adoption	Rigorous toxicological evaluations including long-term animal health and productivity
Underexplored diversity	Potential natural anthelmintics remain overlooked	Broad screening of classes with mechanistic studies (e.g., microbial metabolites)
Poor integration into parasite control programs	Weak practical relevance for farmers	Research on integrated control protocols with natural agents, grazing strategies, and selective treatment

## Data Availability

No new data were created or analyzed in this study. Data sharing is not applicable to this article.
